# Rapid morphologic changes to microglial cells and upregulation of mixed microglial activation state markers induced by P2X7 receptor stimulation and increased intraocular pressure

**DOI:** 10.1186/s12974-021-02251-7

**Published:** 2021-09-20

**Authors:** Keith E. Campagno, Wennan Lu, Assraa Hassan Jassim, Farraj Albalawi, Aurora Cenaj, Huen-Yee Tso, Sophia P. Clark, Puttipong Sripinun, Néstor Más Gómez, Claire H. Mitchell

**Affiliations:** 1 Department of Basic and Translational Science, University of Pennsylvania, 240 S. 40th St, Philadelphia, PA 19104 USA; 2 Department of Orthodontics, University of Pennsylvania, Philadelphia, PA 19104 USA; 3 Department of Preventive Dental Sciences, College of Dentistry, King Saud bin Abdulaziz University for Health Sciences, Riyadh, Saudi Arabia; 4 King Abdullah International Medical Research Center, Riyadh, Saudi Arabia; 5 Department of Ophthalmology, University of Pennsylvania, Philadelphia, PA 19104 USA; 6 Department of Physiology, University of Pennsylvania, Philadelphia, PA 19104 USA

**Keywords:** Microglial M1/M2 activation, P2X7 receptor, Neuroinflammation, Sholl analysis, Chemoattraction migration, P2Y12 receptor, ATP release, Intraocular pressure, Iba1, Glaucoma, Traumatic brain injury

## Abstract

**Background:**

The identification of endogenous signals that lead to microglial activation is a key step in understanding neuroinflammatory cascades. As ATP release accompanies mechanical strain to neural tissue, and as the P2X7 receptor for ATP is expressed on microglial cells, we examined the morphological and molecular consequences of P2X7 receptor stimulation in vivo and in vitro and investigated the contribution of the P2X7 receptor in a model of increased intraocular pressure (IOP).

**Methods:**

In vivo experiments involved intravitreal injections and both transient and sustained elevation of IOP. In vitro experiments were performed on isolated mouse retinal and brain microglial cells. Morphological changes were quantified in vivo using Sholl analysis. Expression of mRNA for M1- and M2-like genes was determined with qPCR. The luciferin/luciferase assay quantified retinal ATP release while fura-2 indicated cytoplasmic calcium. Microglial migration was monitored with a Boyden chamber.

**Results:**

Sholl analysis of Iba1-stained cells showed retraction of microglial ramifications 1 day after injection of P2X7 receptor agonist BzATP into mouse retinae. Mean branch length of ramifications also decreased, while cell body size and expression of *Nos2*, *Tnfa*, *Arg1*, *and Chil3* mRNA increased. BzATP induced similar morphological changes in ex vivo tissue isolated from Cx3CR1^+/GFP^ mice, suggesting recruitment of external cells was unnecessary. Immunohistochemistry suggested primary microglial cultures expressed the P2X7 receptor, while functional expression was demonstrated with Ca^2+^ elevation by BzATP and block by specific antagonist A839977. BzATP induced process retraction and cell body enlargement within minutes in isolated microglial cells and increased *Nos2* and *Arg1*. While ATP increased microglial migration, this required the P2Y12 receptor and not P2X7 receptor. Transient elevation of IOP led to microglial process retraction, cell body enlargement, and gene upregulation paralleling changes observed with BzATP injection, in addition to retinal ATP release. Pressure-dependent changes were reduced in P2X7^−/−^ mice. Death of retinal ganglion cells accompanied increased IOP in C57Bl/6J, but not P2X7^−/−^ mice, and neuronal loss showed some association with microglial activation.

**Conclusions:**

P2X7 receptor stimulation induced rapid morphological activation of microglial cells, including process retraction and cell body enlargement, and upregulation of markers linked to both M1- and M2-type activation. Parallel responses accompanied IOP elevation, suggesting ATP release and P2X7 receptor stimulation influence the early microglial response to increased pressure.

**Supplementary Information:**

The online version contains supplementary material available at 10.1186/s12974-021-02251-7.

## Introduction

The contribution of microglial cells to neural damage is complex, and untangling interactions between stimuli, cell types, and signaling cascades requires a detailed analysis [1]. Microglial cells are resident immune cells in neural tissue responsible for synaptic maintenance and the innate immune response to injury or microbial infiltration [2, 3]. While considered largely beneficial in their immunoquiescent M0 state [4], microglial dysregulation is implicated in several neuroinflammatory pathologies [5–7], including diseases that involve mechanical strain to neural tissue, such as traumatic brain injury, cerebral edema, and glaucoma [8–11]. Identifying signals that link mechanical strain to microglial activation could help reduce the destructive neuroinflammation associated with these disorders.

The release of ATP is a widespread response to mechanical strain found throughout the body, including in neural tissue [12–14]. The released ATP stimulates ionotropic P2X or metabotropic P2Y receptors and can act at adenosine receptors after dephosphorylation [15]. Inflammatory responses are most frequently associated with the P2X7 receptor, and stimulation by ATP can lead to activation of the NLRP3 inflammasome and release of various cytokines linked to neural inflammation [16, 17]. P2X7 receptors are emerging as important regulators of microglial cells, with in vitro experiments in murine microglial cells showing that receptor stimulation leads to release of the IL-1 family cytokines, caspase 1 cleavage, and altered phagocytosis [18, 19].

The mechanical strain accompanying the elevation of intraocular pressure (IOP) is associated with both ATP release [20–22] and microglial activation [23–25]. The P2X7 receptor has been implicated in increased staining for microglial markers CD68 [26] or Iba1 [27] following IOP elevation, but details of the molecular and morphological changes induced in microglial cells by P2X7 receptor stimulation following IOP elevation, and the timing of such responses, remain unclear. In this regard, the particular type of activation state induced in microglial cells by P2X7 receptor stimulation is of particular relevance, as the classical activation state (M1) is traditionally defined by release of proinflammatory cytokines or neurotoxic effectors that exacerbate neurotoxicity [28–30], while the alternative activation states (M2) promote phagocytosis and waste clearance, neurogenesis, axon remodeling, or remyelination after an injury [31–33]. As microglial activation states are now recognized to be more fluid and less binary than suggested by the traditional M1 and M2 classifications [34, 35], a more nuanced understanding of how stimuli modify the morphological and molecular response of microglial cells is needed.

This study provides a detailed analysis of the molecular and morphologic consequences of P2X7 receptor stimulation in microglia cells in vitro and in vivo. The ability of receptor stimulation to trigger process retraction and migration was probed, and effects on markers for M1 and M2 activation states were examined. Receptor stimulation was examined in a more relevant pathophysiological context by elevating IOP. The relationship between IOP elevation, microglial activation, and death of retinal ganglion cells was also investigated. The findings confirm a central role for the P2X7 receptor in the early activation of microglial cells following pressure elevation.

## Materials and methods

### Animal care and use

All procedures were performed in strict accordance with the National Research Council’s “Guide for the Care and Use of Laboratory Animals” and were approved by the University of Pennsylvania Institutional Animal Care and Use Committee (IACUC) in protocol #803584. All animals were housed in temperature-controlled rooms on a 12:12 light:dark cycle with food and water ad libitum. Mice were obtained from Jackson Laboratories (Bar Harbor, ME). B6.129P2(Cg)-CX3CR1^tm1Litt/J^ mice were bred with C57BL/6J mice for pups that were heterozygous for GFP expression on the CX3CR1 promoter (CX3CR1^+/GFP^). Though microglia derived from Cx3CR1^+/GFP^ have an altered transcriptome [36] and physiological responses [37], differences likely represent a more aged phenotype [36], and not a physiologically irrelevant response.

### Intravitreal injections

Intravitreal injections were performed as previously described [38] as detailed in Supplemental Methods.

### Retinal imaging

Dissected retinae were nicked to preserve orientation after enucleation. Retinae were fixed in 4% paraformaldehyde (PFA) for 20 min, incubated with 0.1% Triton X-100 (Sigma-Aldrich) in SuperBlock buffer (Thermo Fisher) for 30 min at 25 °C, then 10% goat serum in SuperBlock for 1 h. Retinal whole mounts or sections were incubated with primary antibodies 18–48 h at 4 °C, with secondary antibodies for 60 min at 25 °C, then mounted with SlowFade Gold anti-fade mounting medium (Molecular Probes). Antibodies are listed in Table 1. For ex vivo experiments, CX3CR1^+/GFP^ mice were euthanized by CO_2_ and enucleated eyes placed into isotonic solution (consisting of (in mM) 105 NaCl, 5 KCl, 6 4-(2-hydroxyethyl)-1-piperazineethanesulfonic (HEPES) acid, 4 Na 4-(2-hydroxyethyl)-1-piperazineethanesulfonic acid, 5 NaHCO_3_, 60 mannitol, 5 glucose, 0.5 MgCl_2_ and 1.3 CaCl_2_, pH 7.4) with or without BzATP and imaged on a Nikon A1R microscope of the University of Pennsylvania Live Cell Imaging Core. Retinal whole mounts were dissected as previously described [38]. Following exposure to BzATP or isotonic solution for 2 h at 37 °C, retinae were fixed with 4% paraformaldehyde for 15 min at 25 °C and mounted using SlowFade Gold medium (Thermo Fisher). Details on the *immunohistochemical approaches*, *image analysis*, *time-lapse of morphological alterations*, and *observer quantification of morphological alterations* are described in detail in Supplemental Material.

**Table 1:** List of antibodies used for immunofluorescent staining

Antibody	Source	Identifier	Dilution	Use
**Primary antibodies**				
Anti-Brn-3a	Santa Cruz	Cat# sc-31984	1:250	Whole mounts
Anti-GFAP	Chemicon	Cat# MAB360	1:500	Immunocytochemistry
Anti-Iba1	Wako	Cat# 019-19741	1:500	Whole mounts, cryosections, immunocytochemistry
Anti-Iba1	Abcam	Cat# AB48004	1:200	Immunocytochemistry
Anti-P2X7 Receptor	Alomone	Cat# APR-008	1:200	Immunocytochemistry
Anti-Synaptophysin	Thermo Fisher	Cat# MA5-14532	1:250	Immunocytochemistry
**Secondary antibodies**				
Anti-Mouse Alexa Fluor 488	Thermo Fisher	Cat# A21202	1:500	Whole mounts
Anti-Rabbit Alexa Fluor 488	Thermo Fisher	Cat# A21206	1:500	Immunocytochemistry
Anti-Mouse Alexa Fluor 488	Thermo Fisher	Cat# A11001	1:500	Immunocytochemistry
Anti-Goat Alexa Fluor 488	Thermo Fisher	Cat# A11055	1:500	Immunocytochemistry
Anti-Rabbit Alexa Fluor 546	Thermo Fisher	Cat# A11035	1:500	Immunocytochemistry
Anti-Goat Alexa Fluor 555	Thermo Fisher	Cat# A21432	1:500	Immunocytochemistry
Anti-Rabbit Alexa Fluor 555	Thermo Fisher	Cat# A31572	1:500	Whole mounts, immunocytochemistry
Anti-Goat Alexa Fluor 555	Thermo Fisher	Cat# A10042	1:500	Cryosections

### Quantitative PCR

Retinae or isolated microglia were homogenized using TRIzol (Invitrogen). RNA was purified with an RNeasy mini kit (Qiagen, Inc.) and converted to cDNA using a High Capacity cDNA Reverse Transcription Kit (Applied Biosystems); qPCR was performed with Power or PowerUp SYBR green (Applied Biosystems) on the 7300 or Quant Studio 3 Real-Time PCR systems (Applied Biosystems) using standard annealing and elongation protocols, with data analyzed using the delta-delta CT approach as described [39]. Primers are listed in Table 2.

**Table 2:** List of qPCR primers

Gene name	GenBank accession	Forward primer (5′–3′)	Reverse primer (3′–5′)	Size (bp)
*Nos2*	NM_010927.4	CCCTTCAATGGTTGGTACATGG	ACATTGATCTCCGTGACAGCC	158
*Tnfa*	NM_013693.3	AAATGGCCTCCCTCTCATCAG	GTCACTCGAATTTTGAGAAGATGATC	73
*Arg1*	NM_007482.3	ACAAGACAGGGCTCCTTTCAG	GGCTTATGGTTACCCTCCCG	148
*Chil3*	NM_009892.3	GAAGGAGCCACTGAGGTCTG	GAGCCACTGAGCCTTCAAC	114
*GAPDH*	NM_017008	TCACCACCATGGAGAAGGC	GCTAAGCAGTTGGTGGTGCA	169

### Microglial cell cultures

Primary retinal microglia were isolated from mouse pups of both sexes P12-P20 using standard methods [40, 41]. Primary brain microglia were also isolated [42]. In both cases, mixed cell cultures were grown in media consisting of high-glucose DMEM (HG-DMEM; Invitrogen) with 10% fetal bovine serum (FBS; Sigma-Aldrich), 1% Penicillin/Streptomycin (Pen/Strep; Gibco), 1% GlutaMAX (Gibco), and 1× MEM with nonessential amino acids (Sigma-Aldrich). Upon confluence, microglia were “shaken off” manually and plated in dishes coated with poly-l-lysine (PLL; Peptides International, 0.01%) and Collagen IV (Corning, 4 μg/ml) in HG-DMEM with 5% FBS, plus 10% of media previously exposed to the mixed cell culture for 5–7 days. Media was changed to DMEM with 5% FBS, 1% Pen/Strep, 1% GlutaMAX, and 1× MEM nonessential amino acids 24 h prior to experimentation.

### Ca^2+^ imaging

Microglia were plated on 25-mm coverslips coated with PLL (0.01%) and Collagen IV (4 μg/ml) and loaded with 10 μM Fura-2 AM (Thermo Fisher) with 0.02% Pluronic F-127 (Thermo Fisher) for 45 min at 37 °C. Cells were washed, mounted in a perfusion chamber, and perfused with isotonic solution without Mg^2+^. Ratiometric measurements were performed using a ×40 objective on a Nikon Diaphot microscope (Nikon) by alternating excitation between 340 and 380 nm wavelengths and quantifying emission ≥ 512 nm with a charge-coupled device camera (All Photon Technologies International) as described [43]. Data were expressed as the ratio of light excited at 340 to 380 nm, F_340/380_, due to complexity of calibration. Statistical comparisons were made by averaging the peak value ± two measurements for all BzATP conditions and the average of the final five measurements for the baseline conditions, including isotonic and A839966 in isotonic solution.

### Microglia migration

Mouse retinal microglia were grown to 80% confluence, detached, resuspended in media outlined above to a concentration of 50,000 cells in 390 μl. After incubation in inhibitors or vehicle for 1 h, Hoechst (1 μg/ml) was added to cells for 10 min. The cell suspension was added to the top wells of a chemotaxis chamber (Neuro Probe) separated from a solution of 1 mM ATP (Sigma-Aldrich) in media by a 10 μm pore filter (Neuro Probe). Cells were allowed to migrate for 3 h, after which unmigrated cells were removed from the top of the filter. Filters were washed in Phosphate Buffered Saline, fixed in 4% PFA, then imaged using the Fluoroskan fluorometer (Thermo Fisher) at 340ex/527em. Background fluorescence was subtracted and data normalized to control. Fluorescence measurements were validated by correlating fluorescence to labeled cell counts from images acquired of the underside of the pore filter.

### Intraocular pressure elevation

A transient controlled elevation of IOP (CEI) procedure was produced in adult mice of both sexes using a modification of the approaches of Morrison [44] and Crowston [45] as previously described [46] to raise IOP to ~  57 mmHg for 4 h. Sustained elevation of IOP was also induced using the microbead injection method as described [47]. Both approaches are detailed in Supplemental Methods.

### Vitreal ATP measurement

Given the difficulties in sampling the small extracellular spaces in the retina without touching cells to trigger mechanosensitive ATP release or rupturing cells to trigger cytoplasmic ATP release, levels in the posterior vitreous were determined. ATP concentration was determined with enucleation soon after IOP returned to baseline after a 4 h elevation and then fast-freezing eyes in dry ice. Eyes were later dissected over dry ice, and vitreal samples were collected by chipping away frozen samples; this prevented intracellular ATP in the cut tissue edges from seeping into the vitreous and contaminating the sample [21]. ATP levels were measured using the luciferin/luciferase assay (Sigma-Aldrich) as described [48].

### Data analysis

Statistical analysis was performed using GraphPad Prism software version 9.0.0 (GraphPad Software, LLC). Significant differences between two unrelated groups were assessed by unpaired Student’s *t* test; paired Student’s *t* tests employed when making comparisons between eyes of the same mouse. For comparisons among three or more groups, one-way analysis of variance (ANOVA) with Dunnet’s multiple comparisons (MC) test was used. For comparisons among groups with two variables, two-way ANOVA followed by Tukey’s multiple comparisons test was applied. For quantification of calcium imaging and sustained IOP measurements, a one-way ANOVA for repeated measures with Sidak’s test for multiple comparisons was used. For Sholl analysis, two-way ANOVA with repeated measures (RM) with Dunnet’s test for MC was used. Results returning *p* < 0.05 were considered significant. Data is represented as mean ± standard deviation, with dots representing individual data points from each retina, sample or image as described.

## Results

### P2X7 receptor stimulation leads to morphologic and molecular activation of microglia in vivo

**Fig. 1:**
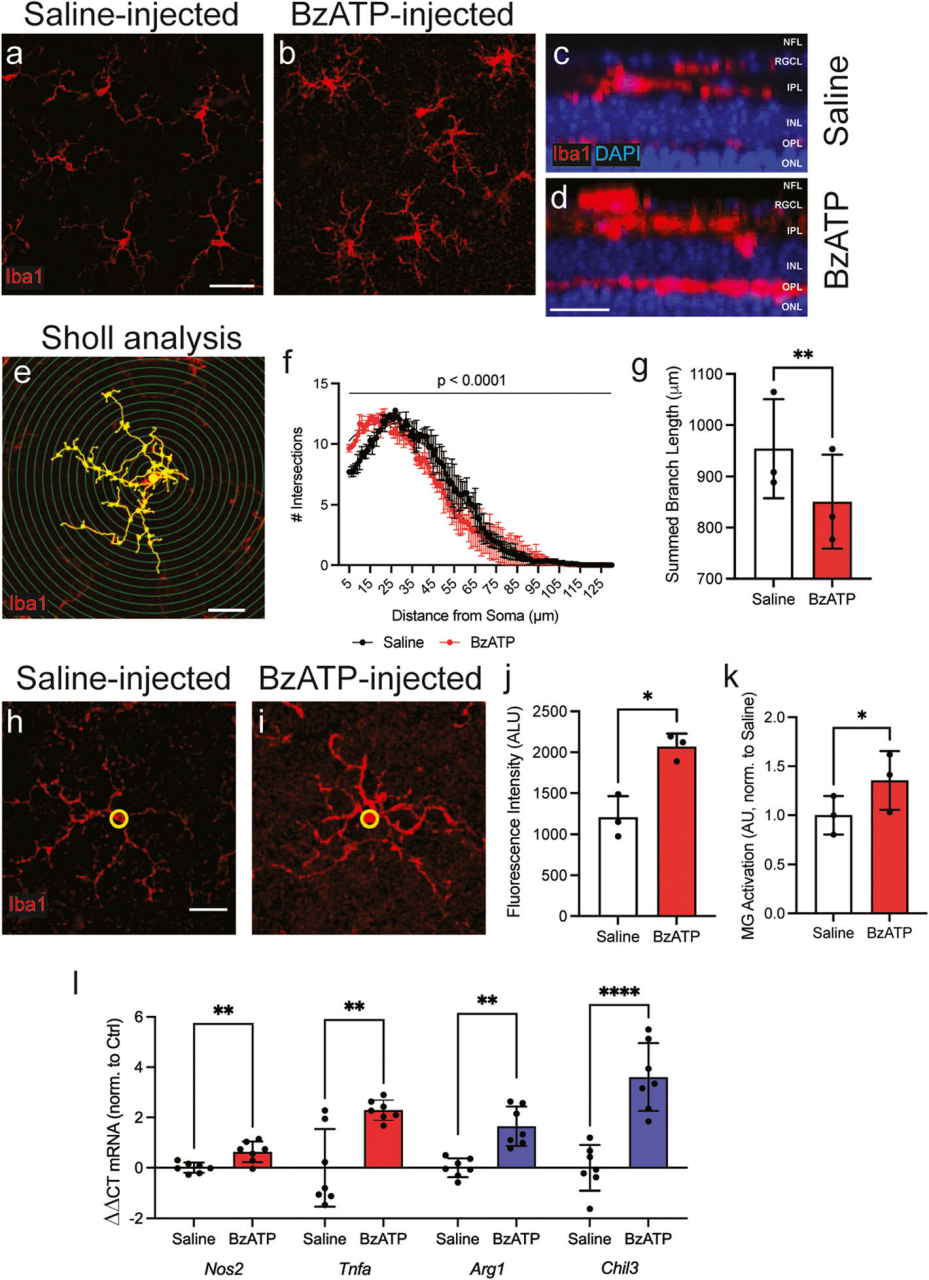
Retinal P2X7 receptor stimulation leads to activated microglia morphology and gene expression. Representative images from retinae fixed 24 h after intravitreal injection with saline (**a**) or 250 μM BzATP (**b**) indicated BzATP exposure led to greater Iba1 expression and altered morphology. **c, d** *Z*-projections of retinal whole mounts demonstrate increased Iba1 staining in the OPL, IPL and RGC layers of the retina with BzATP exposure. **e** Representation of tracing and conversion of Iba1 staining to a binary image for Sholl analysis. **f** Sholl analysis indicated reduced branching complexity of microglia exposed to BzATP (2-way RM ANOVA with Sidak’s MC test; *n* = 3 mice; significance represents interaction value of ANOVA). **g** Summed branch length is reduced in microglia exposed to BzATP as compared to saline (paired Student’s *t* test; *n* = 3 mice). Cell soma size and Iba1 intensity are determined in circled area (yellow ring, 5 μm diameter) surrounding microglia cell body from retinae exposed to **h** saline vs. **i** BzATP. **j** Quantification of Iba1 intensity in circled area (paired Student’s *t* test; *n* = 3 mice). **k** Observer scoring of images taken from saline- or BzATP-exposed retinae showing Iba1-positive microglia are activated with BzATP exposure (paired Student’s *t* test; *n* = 3 mice; normalized to average saline value). **l** Expression of both classical M1- activation genes *Nos2*, *Tnfa*, and alternative M2 activation genes *Arg1*, *Chil3* are elevated in retinae exposed to BzATP (paired Student’s *t* test; *n* = 7 mice)**.** Statistical significance shown at *p < 0.05, **p < 0.01, ****p < 0.0001. Scale bars represent 40 μm (**a**), 15 μm (**d**), and 25 μm (**e, h**)

The response of mouse retinal microglial cells to P2X7 receptor agonist BzATP was investigated in vivo to determine if receptor stimulation was sufficient to evoke morphologic changes. BzATP (250 μM) or saline control was injected intravitreally into eyes of C57Bl/6J mice, and retinae removed after 24 h; this dosage and time course were chosen based on previous trials [38, 49]. Treatment with BzATP led to elevated Iba1 staining and retracted microglial processes, consistent with microglial activation (Fig. 1a, b) [6, 7]. While some degree of increased staining for Iba1 was found throughout the retinal layers (Fig. 1c, d), analysis was focused on the retinal ganglion cell (RGC) and inner plexiform layers (IPL) as the response there was more consistent.

To quantify the extent of process retraction induced by P2X7 receptor stimulation, images were traced to produce binary outputs, and Sholl analysis was performed (Fig. 1e, Supplemental Figure S1a) [50]. Retinal BzATP exposure led to a significant reduction in microglial process length and complexity compared to saline controls (Fig. 1f). Peak intersection distance (Fig S1b) and longest intersection distance (Fig. S1c) were both reduced upon exposure to BzATP. Furthermore, the cumulative length of all branches was reduced by BzATP (Fig. 1g), representing approximately a 10% reduction in summed process length (Fig. S1d). Iba1 intensity was measured in a defined area (5 μm) encircling the microglial soma in randomly selected microglia from saline- or BzATP-injected eyes (Fig. 1h, i). Exposure to BzATP led to a significant elevation of Iba1 immunostaining intensity when compared to control counterparts (Fig. 1j), reflecting a combination of increased soma size and Iba1 expression. Analysis of individual images supported microglial activation in retinae exposed to BzATP (Fig. S1e-i). Sholl analysis and soma staining was supported by evaluation of Iba1 intensity and process retraction by masked observers, with consistent signs of activation across all quadrants in retinae exposed to BzATP (Fig. 1k, S2a-d).

qPCR was used to evaluate the molecular changes accompanying P2X7 receptor stimulation. Genes associated with classical M1 activation, *Nos2* and *Tnfa*, and genes associated with the alternative M2 activation state, *Arg1* and *Chil3*, were elevated in retinae 24 h after in vivo exposure to BzATP (Fig. 1l). Together, the morphological and molecular changes are consistent with microglial activation after injection of P2X7 receptor agonist BzATP.

**Fig. 2:**
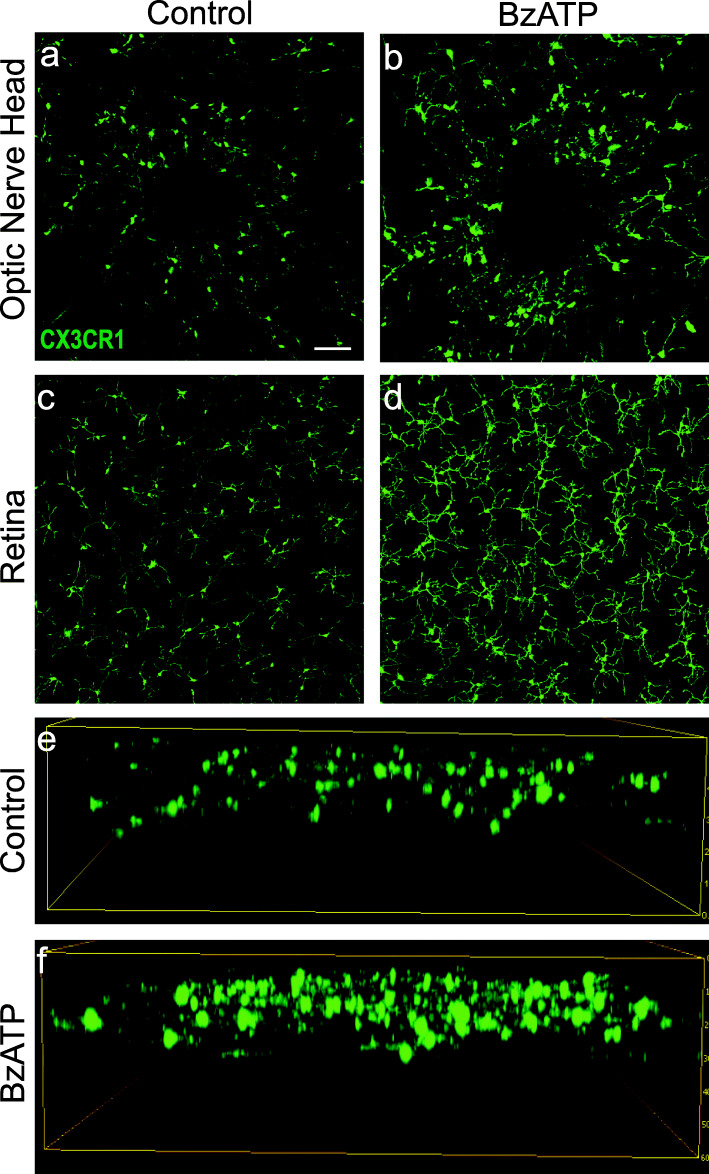
P2X7 receptor stimulation of retina from Cx3CR1^+/GFP^ mice shows activation. **a, b** Retinal whole mounts isolated from Cx3CR1^+/GFP^ mice revealed increased fluorescence at the optic nerve head compared to control media (**a**) after exposure to 200 μM BzATP for 2 h in a petri dish (**b**). This elevation in fluorescence was also seen in the middle nasal areas when comparing control media (**c**) to BzATP exposure (**d**). **e, f** *Z*-projection of middle nasal retinae exposed to control media or BzATP. Bar = 50 μm

The response of microglial cells in isolated ex vivo retinal whole mounts to agonist BzATP was examined to determine if resident microglia were sufficient for the observed increase in staining and alterations in morphology following P2X7 receptor stimulation. Retinal whole mounts derived from heterogeneous mice with a fluorescent tag attached to microglia/macrophage receptor Cx3CR1^+/GFP^ were used to provide an additional method to track changes in microglial morphology. Exposure to BzATP led to a considerable increase in fluorescence, with morphological changes resembling those observed after BzATP injection in vivo (Fig. 2). The increased signal was most noticeable around the optic nerve head (Fig. 2a, b), with prominent cell bodies apparent. Exposure to BzATP also increased the signal throughout the central (Fig. 2c, d) and peripheral retina, and across retinal layers (Fig. 2e, f). This ex vivo response in isolated retina suggests that microglia normally resident within the retina are capable of responding to P2X7 receptor stimulation, although it cannot rule out recruitment of additional monocytes following exposure in vivo.

### P2X7 receptor stimulation leads to morphologic and molecular activation of isolated microglial cells

Isolated microglia cells were examined to determine whether stimulation of the P2X7 receptor could induce effects on microglial cells directly. The relative staining for Iba1 and astrocyte marker GFAP, and neural marker synaptophysin suggested preparations contained > 95% microglial cells (Fig. S3a, b). Similar analysis confirmed identity of brain microglia as shown in more detail recently [51]. To support the microglial identity of the cells, the ability of lipopolysaccharides (LPS) and interleukin-4 (IL-4) to induce expression of activation state markers was determined using qPCR, as these two agonists are traditionally associated with classical and alternative activation states, respectively [28, 52]. Four-hour stimulation of isolated retinal microglial cells with LPS (10 ng/ml) increased expression of *Nos2* and *Tnfa*, while stimulation with IL-4 (10 ng/ml) increased expression of markers for the alternative activation state such as *Chil3* and *Arg1* (Fig. S3c). These responses resemble those recently found for isolated brain microglia [51] and suggest cells cultured under these conditions responded as predicted for microglial cells.

**Fig. 3:**
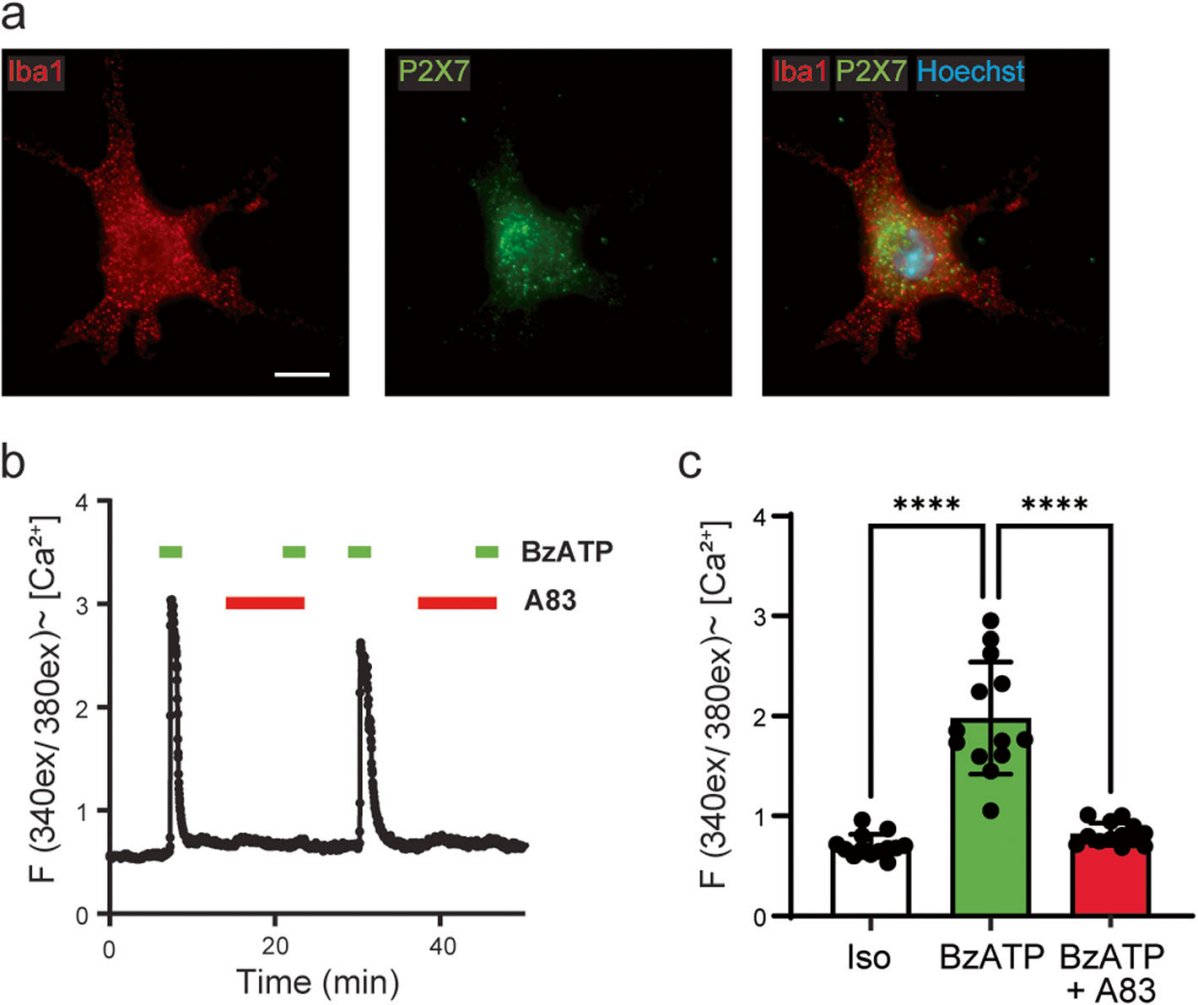
Isolated microglial cells express P2X7 receptors on protein and functional levels. **a** Immunostaining of the P2X7 receptor (green) in primary retinal microglial cells stained positive for Iba1 (red). Bar = 10 μm. **b** Representative trace from a retinal microglia cell loaded with Fura-2 showing elevation in cytoplasmic Ca^2+^ in response to BzATP (100 μM, Mg^2+^ free solution). Data displayed as the ratio of light excited at 340/380 nm, emitted > 520 nm (referred to as “F(340/380)”). The response to BzATP was transiently reduced by 1 μM P2X7 antagonist A839977 (A83). **c** Quantification of the response in multiple cells (RM 1-way ANOVA with Sidak’s MC test; *n* = 12 cells from 3 culture preparations)

Immunocytochemical staining indicated Iba1-positive isolated retinal microglial cells expressed the P2X7 receptor (Fig. 3a). Functional expression of the P2X7 receptor was assessed by examining levels of cytoplasmic Ca^2+^ with the ratiometric indicator Fura-2. A 1-min addition of BzATP raised cytoplasmic Ca^2+^ in the microglial cells (Fig. 3b, c); the response was rapid, with most cells showing a response within 20 s. The response was also reversible upon wash-out of BzATP, and repeatable upon reapplication; these characteristics are consistent with an ionotropic channel with little inactivation like the P2X7 receptor as observed previously [53, 54], and resembled the response reported recently in isolated brain microglia [51]. The P2X7 receptor-specific inhibitor A839977 [55] significantly reduced the Ca^2+^ rise triggered by BzATP, with the robust response to BzATP after removal of the A839977 confirming this decrease (Fig. 3b, c). These responses support the presence of functional P2X7 receptors on these isolated cells, while the responses to IL-4 and LPS (Fig. S3c) supports their characterization as microglial cells.

**Fig. 4:**
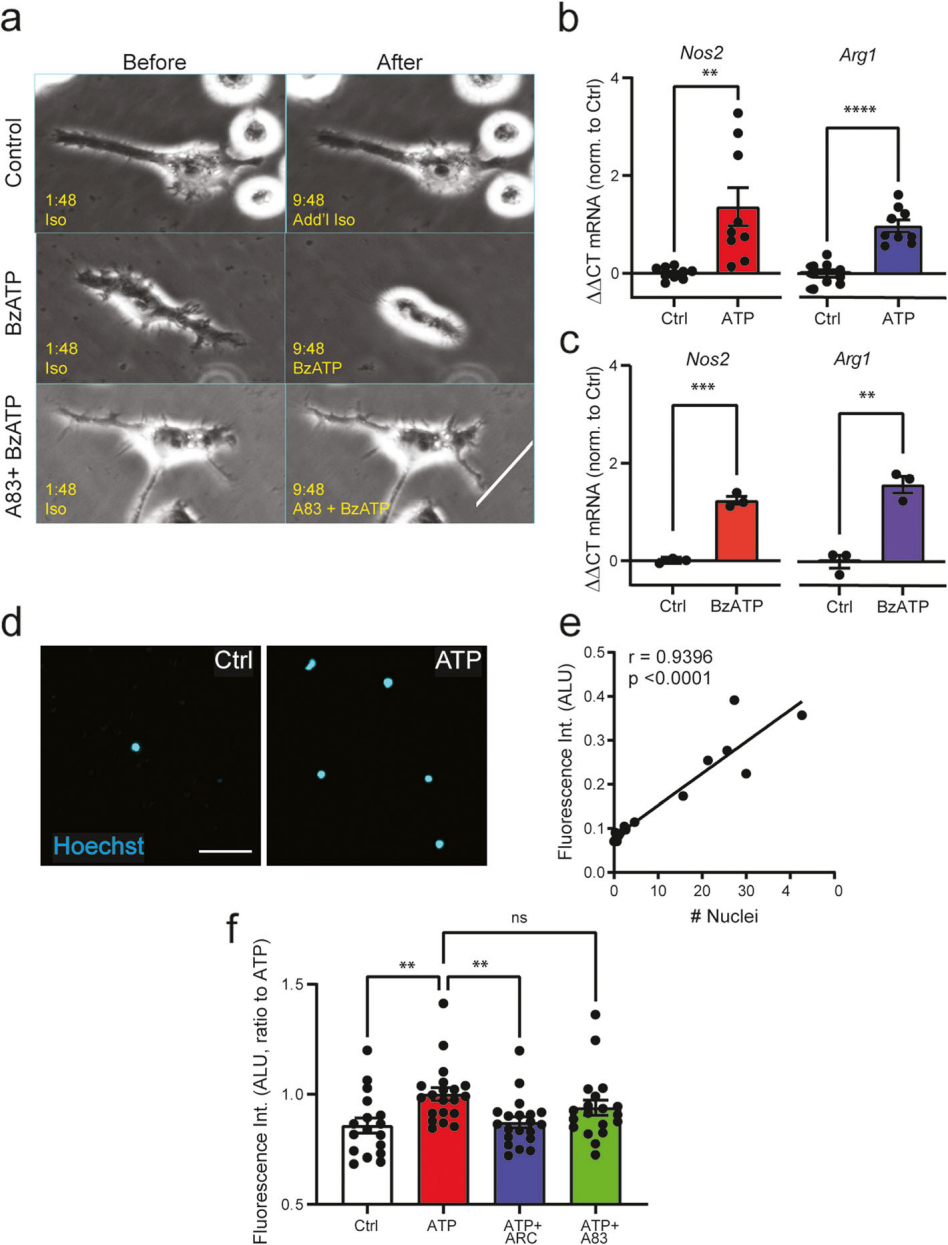
Isolated retinal microglia respond to P2X7 receptor stimulation with rapid retraction, gene activation but not migration. **a** Images of isolated retinal microglial cells taken before (left) and ~ 7min after (right) application of isotonic solution (Control), 250 μM BzATP (BzATP), or 250 μM BzATP (BzATP) ± 10 μM A839977 (A83) suggests P2X7 receptor leads to process retraction in vitro. Similar responses were found in > 7 experiments. Bar = 10 μm, real time in min on image; solution added at minute 3:00; see Fig. S4 for video. Elevated expression of *Nos2* and *Arg1* was detected in cultured retinal microglial cells following 4 h exposure to 1 mM ATP (**b,** *n* = 9 wells from 3 culture preparations) or 200 μM BzATP (**c,** *n* = 3 wells from 1 culture preparation; unpaired Student’s *t* test). **d** Representative images of isolated retinal microglial cells with Hoechst-stained nuclei after passing through a Boyden chamber, indicate that microglia migrate towards a 1 mM ATP gradient. Bar = 50 μm. **e** Correlation between number of Hoechst-stained nuclei in brain microglia per well and fluorescence at 340ex/527em (Pearson’s correlation *r* = 0.9396 with *p* = 0.0001; 1= 17 wells from 1 culture preparation). **f** Migration of retinal microglia towards 1 mM ATP was inhibited by exposure to 10 μM P2Y12 inhibitor AR-C 69931 (ARC) in the presence of ATP but not 1 μM A839977 (A83) in the presence of ATP. (1-way ANOVA with Sidak’s MC test; *n* = 17 Ctrl, 20 ATP, 20 ARC, 19 A83 wells from 4 culture preparations). Statistical significance shown as **p < 0.01, ***p < 0.001, ****p < 0.0001, ns = not significant

The effect of BzATP on morphology of isolated microglia was examined to determine whether changes observed in vivo could be replicated in the absence of other cell types. BzATP triggered a retraction of microglial processes and a rounding of the cell body in greater than 75% of observed cells (Fig. 4a). This response was rapid, starting less than 7 min after BzATP application; additional movie files show this in more detail (Fig. S4). The effect of BzATP on microglial morphology was greatly reduced in the presence of inhibitor A839977, supporting action of BzATP at the P2X7 receptor. This suggests that stimulation of the P2X7 receptor acting directly on microglial cells was sufficient to trigger the rapid morphological changes seen in vivo.

Stimulation of the P2X7 receptor on isolated microglial cells also induced changes in gene expression with parallels to those observed in vivo after P2X7 receptor stimulation. Specifically, the endogenous agonist ATP (Fig. 4b), and P2X7 receptor agonist BzATP (Fig. 4c) both increased expression of *Nos2* and *Arg1*.

To determine if the effects of P2X7 receptor stimulation on microglial process retraction extended to migration, chemoattraction of isolated retinal microglial cells to ATP was evaluated using a 2-part Boyden Chamber. Imaging of the filter with bound microglia confirmed elevated migration of retinal microglia towards an ATP concentration gradient (Fig. 4d). Measurements indicated that the number of Hoechst-stained microglial cells closely reflected total Hoechst fluorescence (Fig. 4e), with migration levels optimal 3 h after the addition of cells to the chamber. Microglial migration towards 1 mM ATP was inhibited by inclusion of P2Y12 receptor inhibitor AR-C 69931 with the ATP, but not inclusion of P2X7 receptor inhibitor A839977 with the ATP (Fig. 4f), suggesting the effects of P2X7 receptor stimulation are focused on retraction of ramifications and not the actual migration of retinal microglia.

### Role of ATP and the P2X7 receptor in pressure-dependent microglial activation

**Fig. 5:**
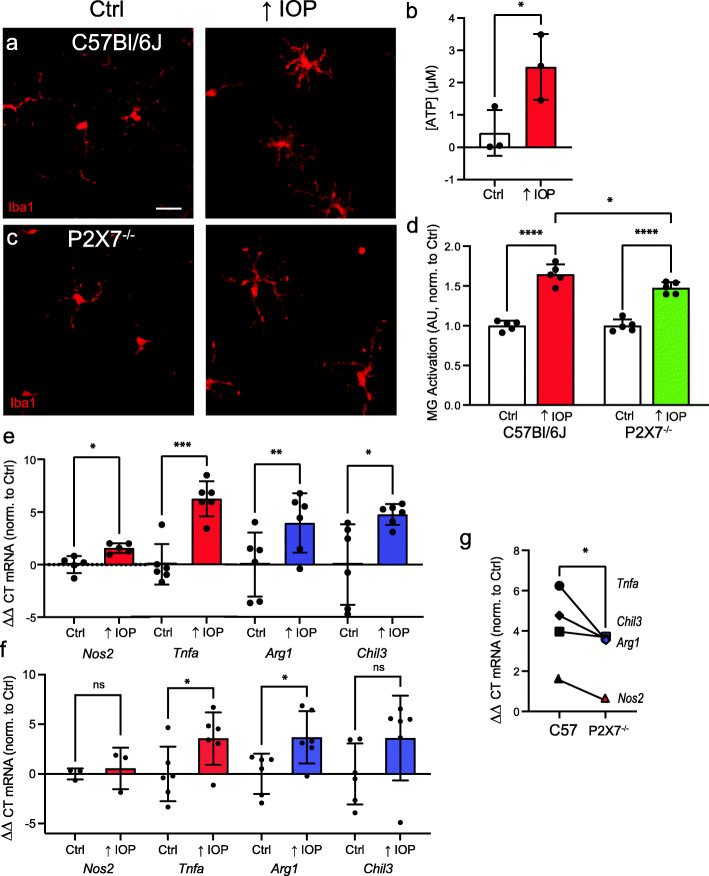
Transient elevation of IOP releases ATP and activates microglia through P2X7 receptor involvement. **a** Representative image of staining for Iba1 in a retinal whole mount from an unpressurized C57Bl/6J mouse eye (Ctrl, left) and from an analogous region 20 h after elevation of IOP to 57 mmHg for 4 h (right). Retinal microglia subject to IOP elevation showed increased soma size, increased staining for Iba1, and shorter, thicker projections. Bar = 20 μm. **b** Increase in ATP concentration of posterior vitreous humor after elevation of IOP (paired Student’s *t* test; retinal pairs from 3 mice). **c** Iba1 staining from a P2X7^−/−^ mouse indicates reduced morphological activation after elevation of IOP (right) when compared to unpressurized retina (left). **d** Quantification of morphological activation of microglia across central and middle regions suggests IOP elevation triggered greater morphological activation in C57Bl/6J than P2X7^−/−^ retinae (2-way ANOVA with Tukey’s MC test; dots are *n* = 10 retinae from 5 mice per C57Bl/6J, P2X7^−/−^ strain; data normalized to unpressurized score for each strain). **e** qPCR showing increased expression of *Nos2*, *Tnfa*, *Arg1*, and *Chil3* in the retina after elevation of IOP in C57Bl/6J (**e**) mice. Dots represent change in expression from a single mouse, with expression normalized to the average ΔΔCT value of unpressurized contralateral eyes (paired Student’s *t* tests; *Nos2: n* = 5 mice, *Tnfa*, *Arg1*, *Chil3: n* = 6 mice). **f** A small but significant increase was observed in retinal gene expression of *Arg1*, and *Tnfa*, but not *Nos2* or *Chil3* after IOP elevation in P2X7^−/−^ mice (Paired Student’s *t* tests; *Nos2 n* = 6 retinae from 3 mice, *Tnfa*, *Arg1*, *Chil3*, *n* = 12 retinae from 6 mice). **g** Relative change in retinal expression of key genes after elevation of IOP in C57Bl/6J mice compared to P2X7^−/−^ mice. Values represent mean ΔΔCT levels for each gene compared to unpressurized control retinae (unpaired Student’s *t* test; *n* = 5 genes). Statistical significance shown as *p < 0.05, **p < 0.01, ***p < 0.001, ****p < 0.0001, ns = not significant

Given that increased IOP is associated with both ATP release and microglial activation, experiments investigated the role of the P2X7 receptor in the morphological and molecular changes induced by IOP elevation. The effect of moderate transient IOP elevation on microglia cells was examined by increasing IOP to 57.0±0.4 mmHg for 4 h, with retinal whole mounts fixed 24 h later and stained for Iba1; previous studies indicated robust inflammatory responses at this point [46]. Iba1 staining revealed a noticeable change in microglial morphology in eyes exposed to elevated IOP, with larger cell bodies and shorter processes in retinal tissue exposed to elevated IOP as compared to control (Fig. 5a). As mechanosensitive release of ATP is an early and sustained event found after pressure elevation in bovine, mouse, rat, and primate eyes [21, 46, 56], ATP concentration was determined as soon as pressure returned to baseline in the transient model. ATP levels sampled in the vitreal humor near the inner limiting membrane were significantly elevated in eyes subjected to increased IOP as compared to normotensive controls (Fig. 5b). Given the presence of elevated ATP, the response to IOP elevation was examined in retinae from P2X7^−/−^ mice. Microglia cells from P2X7^−/−^ mice showed a smaller change in morphology after transient IOP elevation (Fig. 5c). Histological quantification confirmed a clear increase in the morphological signs of activation in microglial cells from retinae of C57Bl/6J retinae following IOP elevation, and reduced activation in retina from P2X7^−/−^ mice (Fig. 5d).

The pattern of gene change following transient IOP elevation was similar to that found after BzATP injection, with elevation of classical activation markers *Nos2* and *Tnfa*, and genes associated with the alternative activation state, *Arg1* and *Chil3* (Fig. 5e). The expression of genes *Tnfa* and *Arg1* increased in retina of P2X7^−/−^ mice after exposure to elevated IOP, but the rise was smaller and there was no significant change in *Nos2* or *Chil3* expression (Fig. 5f). Overall, the change in expression of genes following IOP elevation was reduced in retina from P2X7^−/−^ mice as compared to C57Bl/6J mice (Fig. 5g). Altogether, both morphological and molecular changes following transient IOP elevation are consistent with a contribution from the P2X7 receptor towards activation.

**Fig. 6:**
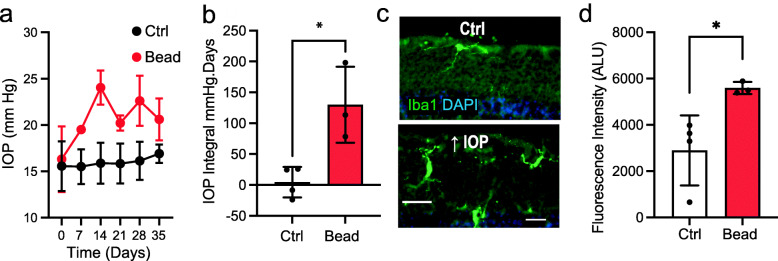
Microglial activation in retina from a model of sustained ocular hypertension. **a** Weekly IOP measurements from mice injected with magnetic beads (red) or saline control (black; 1-way RM ANOVA with Sidak’s MC test). **b** The IOP integral (right) expressed as summed mmHg days exposure over baseline IOP, for bead and saline-injected eyes (unpaired Student’s *t* test). **c** Representative staining for Iba1 in cryosections 7 weeks after injection with saline (top) or beads (bottom), suggesting sustained elevation of IOP also induces changes in the microglial phenotype emblematic of activation. Bar = 50 μm. **d** Quantification of a 5-μm area surrounding the soma indicates significant elevation of Iba1 intensity per cell in bead-injected mice (unpaired Student’s *t* test). All data from Ctrl: *n* = 4 retinae from 3 mice, Bead: *n* = 3 retinae from 3 mice. Statistical significance shown as *p < 0.05

As glaucoma is primarily a chronic disorder, responses were examined in retinae from C57Bl/6J mice subjected to sustained elevation of IOP via magnetic bead blockage of aqueous humor outflow (Fig. 6a,b). Similar morphological differences were observed in Iba1-stained cells in the sections from central retinal regions of mice with bead injection, including retraction of processes, cell soma swelling, and increased expression of Iba1 when compared to saline-treated retinae (Fig. 6c). Quantification of Iba1 intensity in the soma area showed significant elevation with sustained IOP elevation (Fig. 6d)

### Retinal ganglion cell loss, microglial activation, and the P2X7 receptor

**Fig. 7:**
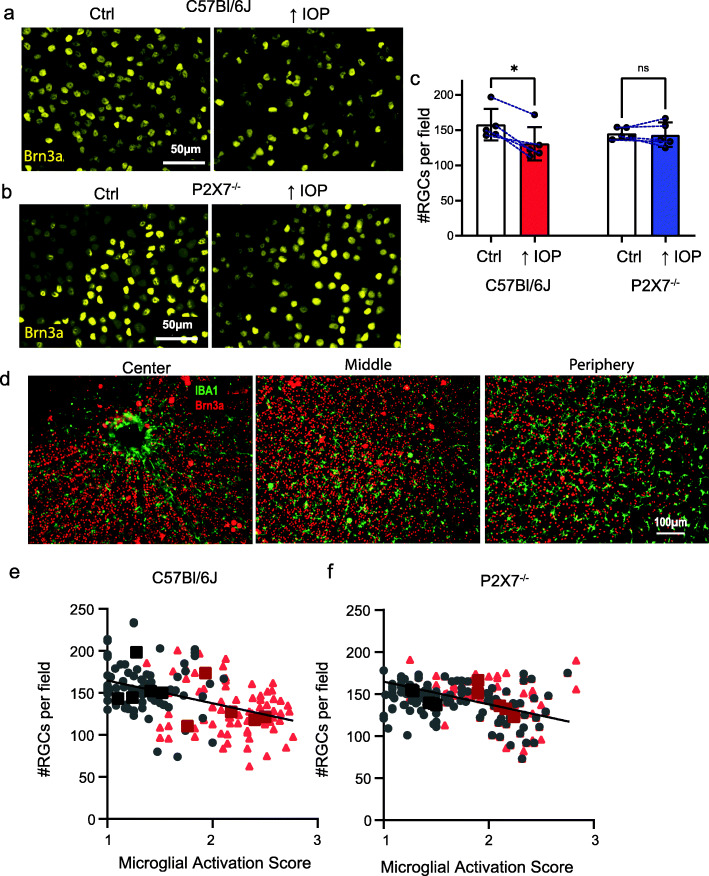
Ganglion cell death and microglial activation. **a** Representative images show that staining for RGC marker Brn3a is decreased in retinae 1 day after a 4-h IOP elevation (right) compared to unpressurized C57Bl/6J mice (Ctrl; left). **b** A decrease in Brn3a staining was not observed in retinae from P2X7^−/−^ mice after IOP elevation as compared to unpressurized control eyes. **c** There was a significant decline in Brn3a-labeled RGCs in retinae exposed to elevated IOP compared to normotensive controls from C57Bl/6J eyes, but not P2X7^−/−^ mice (*p = 0.0017, paired *t*-test with line connecting normotensive and elevated IOP from same mouse, *n* = 5 mice, mean of ~ 24 images per eye). **d** Retinal whole mount from a C57Bl/6J mouse showing the spatial relationship between RGCs stained with Brn3a (red) and microglia stained with Iba1 (green); images show staining across the central region with the optic nerve head (left), the middle region (center), and peripheral areas (right), of the retina. Relationship between RGC number and microglial activation score for C57Bl/6J (**e**) and P2X7^−/−^ mice (**f**) under normotensive (gray) and elevated IOP (red) conditions. Small symbols from individual images (up to 24 per eye) while larger squares represent mean values per eye (*n* = 5 per condition). Lines are linear regression fit to image data (C57Bl/6J: F = 37.70, (1,155) *p* < 0.0001, *R*^2^ = 0.196; P2X7^−/−^ F = 8.22, (1,152), *p* = 0.0047, *R*^2^ = 0.05)

The relationship between microglial activation, the P2X7 receptor, and retinal ganglion cell loss was examined to better understand the consequences of microglial activation to ganglion cell health. Retinal whole mounts used above were co-stained for the ganglion cell transcription factor Brn3a and the number of cells present in each of the 24 regions (see Fig. S1i) were counted. In C57Bl/6J mice, IOP elevation led to a modest reduction in Brn3a-positive cells as compared to normotensive eyes (Fig. 7a), although little change was seen in P2X7^−/−^ mice (Fig. 7b). Quantification confirmed a significant decline in the number of Brn3a-positive cells with IOP elevation in C57Bl/6J mice, but not P2X7^−/−^ mice (Fig. 7c). Close overlap between microglial and retinal ganglion cells occurs throughout the retina (Fig. 7d), although quantification indicated the ratio of microglia to RGCs differed in peripheral regions as compared to central and middle areas (Fig. S5a), supporting the focus on these regions. Analysis of activation from individual images across retinal regions supports these findings for microglial and retinal ganglion cell quantification (Fig. S5b, c).

The microglial activation score was compared to ganglion cell number in all images in the central and middle areas for C57Bl/6J mice (Fig. 7e) and P2X7^−/−^ mice (Fig. 7f). Images with fewer ganglion cells tended to show greater microglial activation; this relationship was greater for C75Bl/6J mice (slope − 27.0) than for P2X7^−/−^ mice (slope − 10.7). The correlation between microglial activation score and ganglion cell number was closer for C75Bl/6J mice with a Pearson’s *r* of 0.442, vs 0.227 for P2X7^−/−^ mice, although considerable variability across the eyes and between mice of both strains limited the strength of the overall correlation.

## Discussion

The data presented in this manuscript illustrate several complex consequences of P2X7 receptor activation in microglial cells. Detailed morphological analysis indicated that administration of P2X7 receptor agonist BzATP to murine retina in vivo reduced branch length, and increased soma size and Iba1 expression in microglial cells, emblematic of microglia activation. Furthermore, retinal exposure to BzATP led to gene expression upregulation of *Nos2*, *Tnfa*, *Arg1*, and *Chil3*, associated with microglial activation into a mixed classical (M1)/alternative (M2) state. Ex vivo retinal explants demonstrated comparable morphological changes following exposure to BzATP. Isolated retinal microglial cells displayed parallel molecular changes after exposure to ATP or BzATP in vitro, with upregulation of classical activation marker *Nos2* and alternative activation marker *Arg1*. Transient elevation of IOP led to similar morphological changes, with larger cell bodies and shorter processes, and increased expression of *Nos2*, *Tnfa*, *Arg1*, and *Chil3.* However, both morphological and molecular changes were reduced in retinae from P2X7^−/−^ mice. The similarities in the morphological and molecular changes induced by BzATP and elevated IOP, combined with the reduced responses in P2X7^−/−^ mice to IOP elevation, implicate the P2X7 receptor in some of the early inflammatory responses to increased pressure in the retina.

Several observations strengthen the findings of this study. (1) The convergence of morphological evidence from in vitro and in vivo experiments supports the validity of conclusions, with rapid retraction of microglial extensions within moments of BzATP application to isolated cells complementing the detailed Sholl analysis in vivo; (2) Convergent molecular data from in vitro and in vivo experiments imply P2X7 receptor stimulation upregulates markers traditionally associated with both M1 and M2 activation states; (3) Parallel morphological effects of BzATP on retinal explants imply resident retinal microglial cells were sufficient to produce this response without recruitment of external cells; (4) While BzATP can act at other purinergic receptors, use of P2X7^−/−^ mice, combined with antagonist A839977 (IC_50_ 150 nm, [57]) and immunohistochemical identification strongly implicates the P2X7 receptor; (5) Boyden chamber studies suggest the P2X7 receptor effect is focused on retraction of microglial ramifications and not on chemoattractant migration; (6) Use of models for both transient and sustained elevation of IOP, combined with live-cell imaging, strengthen understanding of the time course of the response to P2X7 receptor stimulation. Overall, the data considerably extend our understanding of the P2X7 receptor, microglial activation, and increased pressure.

### P2X7 receptor and microglial activation states

The data above support the theory that the molecular response to transient P2X7 stimulation represents a mixed M1/M2 microglial cell state. A 4-h stimulation of the P2X7 receptor in cultured microglia with 1 mM ATP or 200 μM BzATP upregulated classical activation gene marker *Nos2* and alternative activation gene marker *Arg1* (Fig. 4b,c), paralleling upregulation of classical activation markers *Nos2* and *Tnfa* and alternative activation markers *Arg1* and *Chil3* with in vivo administration of BzATP, or elevation of IOP (Fig. 1l; Fig. 5e). P2X7 receptor stimulation has been shown to lead to upregulation of classical activation markers in a variety of inflammatory cell types [58–60], with a study on the SOD-G93A model of amyotrophic lateral sclerosis also suggesting upregulation of alternative activation markers [61]. Given the emerging role of the P2X7 receptor in modulating phagocytosis, autophagy, and lysosomal clearance by microglial cells [51], this mixed activation may have important implications for the health of aging tissues. Previous studies utilizing acute elevation of IOP in rats have focused on neurotoxic cytokine release from microglia [26]. However, directing microglia away from neuroinflammatory states is a putative therapeutic for conditions like brain trauma that are accompanied with transient ATP elevation [62–64]. A full understanding of putative anti-inflammatory therapies for glaucoma and their effects on microglia, such as P2X7 receptor inhibition [65, 66], carbon monoxide [67, 68], antibiotics [69, 70], or downstream effectors [10] will help elucidate the role classical and alternative activation pathways play in retinal degeneration.

### Microglial activation as an early event in retinal degeneration

The findings above add relevant details to a growing body of evidence implicating microglial activation in various retinal degenerations including glaucoma. Activated microglia and associated inflammatory markers TNF-α and NOS-2 have been detected at the optic nerve head in human glaucomatous eyes [71, 72]. Morphological changes to microglial cells were detected in 3-month-old DBA/2J mice, prior to detection of neural damage [7], and documented 3 days after induction of ocular hypertension in rats [27, 73]. Purinergic involvement in the microglial response was suggested by the ability of purinergic receptor blocker OxATP to inhibit the rise in CD68 expression in rat retina following transient elevation of IOP to 90 mmHg [26]. Qualitative analysis also suggestsed antagonist Brilliant Blue G reduced staining of Iba1 following injection of hypertonic saline into rat episcleral veins [27]. The current study indicates the microglial response occurs early, with activation found 24 h after BzATP administration (Fig. 1), 24 h after a 4-h elevation of IOP (Fig. 4), and with retraction occurring within minutes of receptor stimulation in vitro (Fig S4). Within this context, the inability of P2X7 antagonist A839977 to significantly prevent microglial migration (Fig. 4f) distinguishes process retraction from cell migration; whether the P2X7 receptor interacts with THIK-1 channel recently implicated in microglial surveillance remains to be tested using sufficiently high agonist levels [74].

The relationship between microglial activation and retinal ganglion cell loss is complex. Detailed analysis of microglial activation and surviving ganglion cell number indicates an inverse correlation following IOP elevation which was reduced in P2X7^−/−^ mice (Fig. 7). While it is tempting to assume this implicates microglial activation in ganglion cell death via the P2X7 receptor, several observations urge caution. For example, the variation among the 24 images analyzed per retina and between mice was considerable, weakening the correlation. In addition, stimulation of the P2X7 receptor can directly kill retinal ganglion cells [38], while ganglion cell death can itself lead to microglia activation [4]. Activation of A1 astrocytes has been implicated as a key step in pressure-related ganglion cell death [75, 76], and the geometric relationship between astrocytes, microglial cells, ganglion cell soma, and ganglion cell axons is complex, especially as the axons are thought to be a primary target in glaucomatous pathology [77, 78]. P2X7 receptor inhibition has been implicated as a therapeutic strategy for several retinal diseases including age-related macular degeneration [65, 79], diabetic retinopathy [80–83], and glaucoma [49, 66, 84–86]. While topical administration of a P2X7 receptor antagonist preserved general RGC function in the DBA2/J model [66], recent work suggests the P2X7 receptor also has beneficial effects on retinal ganglion cells after moderate IOP elevation [87], and can trigger the release of neuroprotective cytokine IL-3 [88]. These complex responses may also reflect the mixed microglial activation states identified in the present study. The precise relationship between microglial activation, ganglion cell death, and the P2X7 receptor is likely context dependent and awaits future clarification.

## Conclusion

In summary, our results support a model whereby P2X7 receptor stimulation alone is sufficient to cause microglial activation and that this activation occurs rapidly after receptor stimulation or following ATP release with elevation of intraocular pressure. Furthermore, although the P2X7 receptor is traditionally associated with its proinflammatory role [58], P2X7 receptor stimulation here led to a mixed activation state in microglial cells, suggesting the response is complex. Microglia activation was increased with elevated IOP, reduced in P2X7^−/−^ mice and was loosely associated with loss of retinal ganglion cells following increased IOP. The impact of the mixed M1/M2 activation state on the response to P2X7 receptor stimulation, and the corresponding influence on both inflammation and phagocytosis/clearance needs to be determined [51]. As P2X7 receptor modulation is being targeted for retinal disorders [65], the quantitative approaches used in this study can add to a deeper understanding of P2X7 receptor signaling and any putative beneficial effects.

## Supplementary Information

**Additional file 1 **: **Supplementary Figure S1. Details of effect of intravitreal injection of BzATP on Sholl and other image analysis. a** Representations of i- Iba1 staining; ii- image tracing, iii - concentric rings placed every 5 μm for analysis of crossing and length; and iv - conversion to a binary image. Bar = 25 μm. **b, c** Tracings were analyzed for distance from soma at which peak number of intersections with concentric rings (**b**) and farthest intersection with concentric ring (**c**) occurred (paired Student’s t-test; n = 6 retinae of 3 mice). **d** Total summed branch length averaged per mouse was reduced by approximately 10% (paired Student’s t-test; n = 6 retinae of 3 mice; Normalized to mean saline injected = 1.0000, mean 250 μM BzATP = 0.8914). **e** Sholl analysis performed with individual cells (2-way Repeated Measures ANOVA with Sidak’s Multiple Comparison’s Test; n = 41 Saline, 46 BzATP) cells from 6 retinae of 3 mice; significance represents Multiple Comparison’s, data measured every 5 μm). **f**, **g** Absolute (**f**) and normalized (**g**, to avg saline) of Sholl data from individual images confirms reduction in branch length of microglia exposed to BzATP (unpaired Student’s t-test; n = 41 Saline, 46 BzATP cells from 6 retinae of 3 mice; Normalized mean saline = 0.9941, mean BzATP = 0.8930). **h** Quantification of Iba1 intensity in 5 μm ring around soma of individual cells from 6 retinae from 3 mice (unpaired Student’s t-test; n = 60 saline, 55 BzATP cells from 6 retinae of 3 mice). i. Schematic diagram indicating approximate placement of images analyzed for microglia and RGCs. *p < 0.05, **p < 0.01, ****p < 0.0001. **Figure S2. Validation of observer scoring method of morphological microglial activation. a** Representative **i**mages derived from Iba1-immunostained retinal whole mounts indicating a score of 1, 2, or 3. Bar = 50 μm. **b** A significant correlation between observer scoring and microglial Iba1-soma intensity was found. Observer scoring was normalized to the mean of 3 saline injected mice, while soma intensity was quantified in a 5 μm diameter ring as described in Figure 1. Each dot represents the mean value among masked, trained observers (Pearson’s correlation r = 0.6882 with p = 0.0006; n = 21 images, 6 retinae from 3 mice). **c** Scoring of microglial activation shows homogeneity between trained observers. **d** Observer scoring of images taken from saline- or BzATP-exposed retinae analyzed by image (unpaired Student’s t-test; n = 12 saline, 9 BzATP images, from 6 retinae of 3 mice). **p < 0.01. Bar = 50 μm. **Figure S3. Confirmation of cellular identity of isolated microglial cells. a** Immunocytochemistry indicating absence of glial marker GFAP (top) or neuronal marker synaptophysin (Synapt, bottom) in primary cultures of retinal microglial cells. Bar = 20 μm. **b** Isolated brain microglial cells stained for Iba1 but not GFAP. Bar = 20 μm. **c** qPCR results from cultured retinal microglial cells exposed for 4 hrs to DMSO (Ctrl), 10 ng/ml LPS (LPS), or 10 ng/ml IL-4 (IL4); changes in relative expression of mRNA for *Nos2*, *Tnfa*, *Arg1*, *and Chil1* were consistent with microglial cell polarization. (n = 6-9 samples from 2 to 3 biological replicates). **Figure S4.** Videos demonstrating microglial retraction with exposure to BzATP. 15 minute videos were recorded at 15 frames per second, with all solutions added at t = 3 min. **a** Addition of fresh control solution led to few morphological changes. **b** Addition of 250 μM BzATP resulted in rapid retraction of microglial extensions and rounding of cells. **c** In the presence of 10 μM A839977 (and following 30 min preexposure to 10 μM A839977), 250 μM BzATP induced little retraction. All solutions Mg^2+^-free to prevent block of the P2X7 receptor. **Figure S5. a** RGCs (left) and microglia (right) cell numbers were counted per image and averaged within superior inferior, nasal, or temporal regions. There was a significant difference in distribution of RGCs in peripheral retinal areas (RM 1-way ANOVA with Sidak’s MC test; n = 12 regions from 3 mice). **b** Quantification of morphological activation from individual images of microglia across central and middle regions suggests greater activation in C57Bl/6 J retinae than in P2X7^−/−^ retinae (2-way ANOVA with Tukey’s MC test; n = 52 regions from 10 retinae derived from 5 mice per C57Bl/6 J, P2X7^−/−^ strain; data normalized to unstimulated score for each strain). **c** RGC numbers counted in central and middle fields under normotensive (Ctrl) and increased IOP in mice from both genotypes. Statistical significance shown as ** *p* < 0.01, ***p < 0.001, ****p < 0.0001, ns = not significant.

**Additional file 2.** : Supplemental Methods

## Data Availability

All data generated or analyzed during this study are included in this published article and its supplementary information files, or are available from the corresponding author upon reasonable request.

## Abbreviations

ATP: Adenosine triphosphate
BzATP: Benzoylbenzoyl-ATP
Ctrl: Control
Cx3CR1: CX3C chemokine receptor 1
ELISA: Enzyme-linked immunosorbent assay
GFAP: Glial fibrillary acidic protein
Iba1: Ionized calcium-binding adapter molecule 1
IL-1β: Interleukin 1 β
IL-4: Interleukin 4
IPL: Inner plexiform layer
IOP: Intraocular pressure
LPS: Lipopolysaccharides
NLRP3: NOD-, LRR- and pyrin domain-containing protein 3
OPL: Outer plexiform layer
PLL: Poly-l-Lycine
RGC: Retinal ganglion cell
