# Supplementary material for: Rapid morphologic changes to microglial cells and upregulation of mixed microglial activation state markers induced by P2X7 receptor stimulation and increased intraocular pressure
Source: J Neuroinflammation. 2021 Sep 20;18:217. doi: 10.1186/s12974-021-02251-7 (PMC8454080; doi:10.1186/s12974-021-02251-7)

Supplemental Figure 1

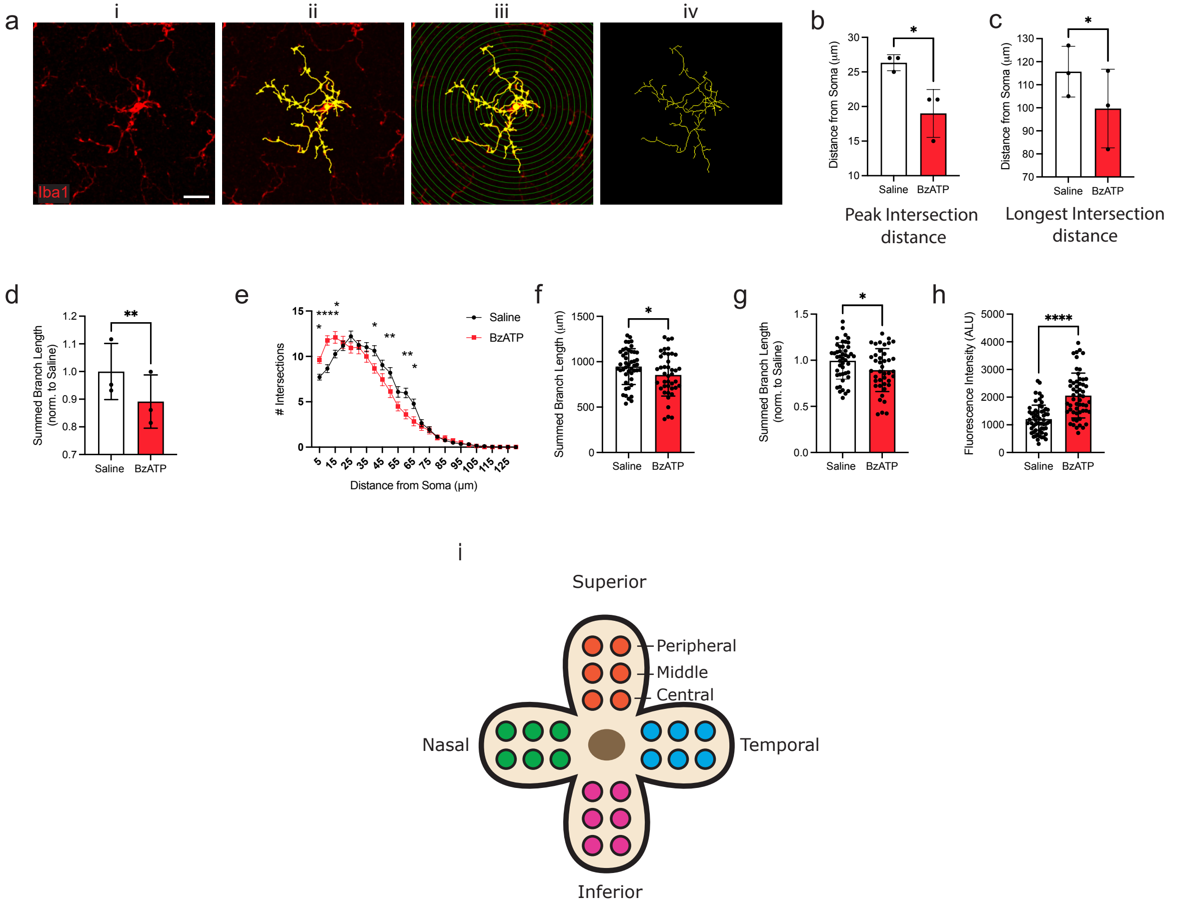

Supplementary Figure 2.

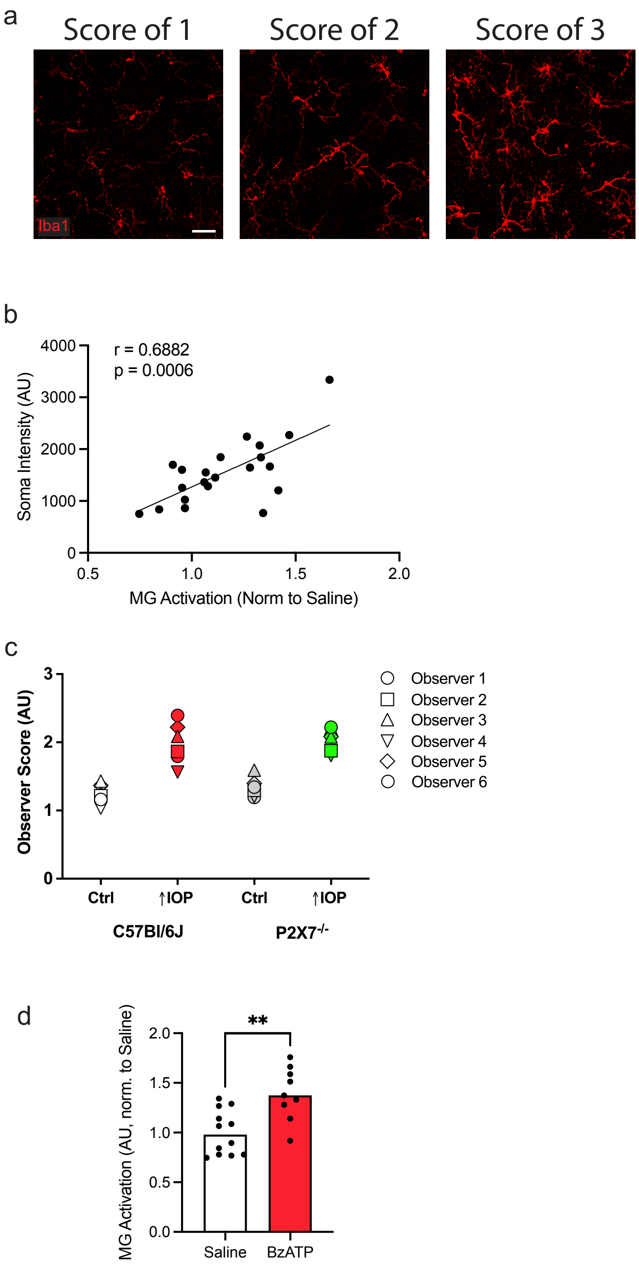

Supplementary Figure 3

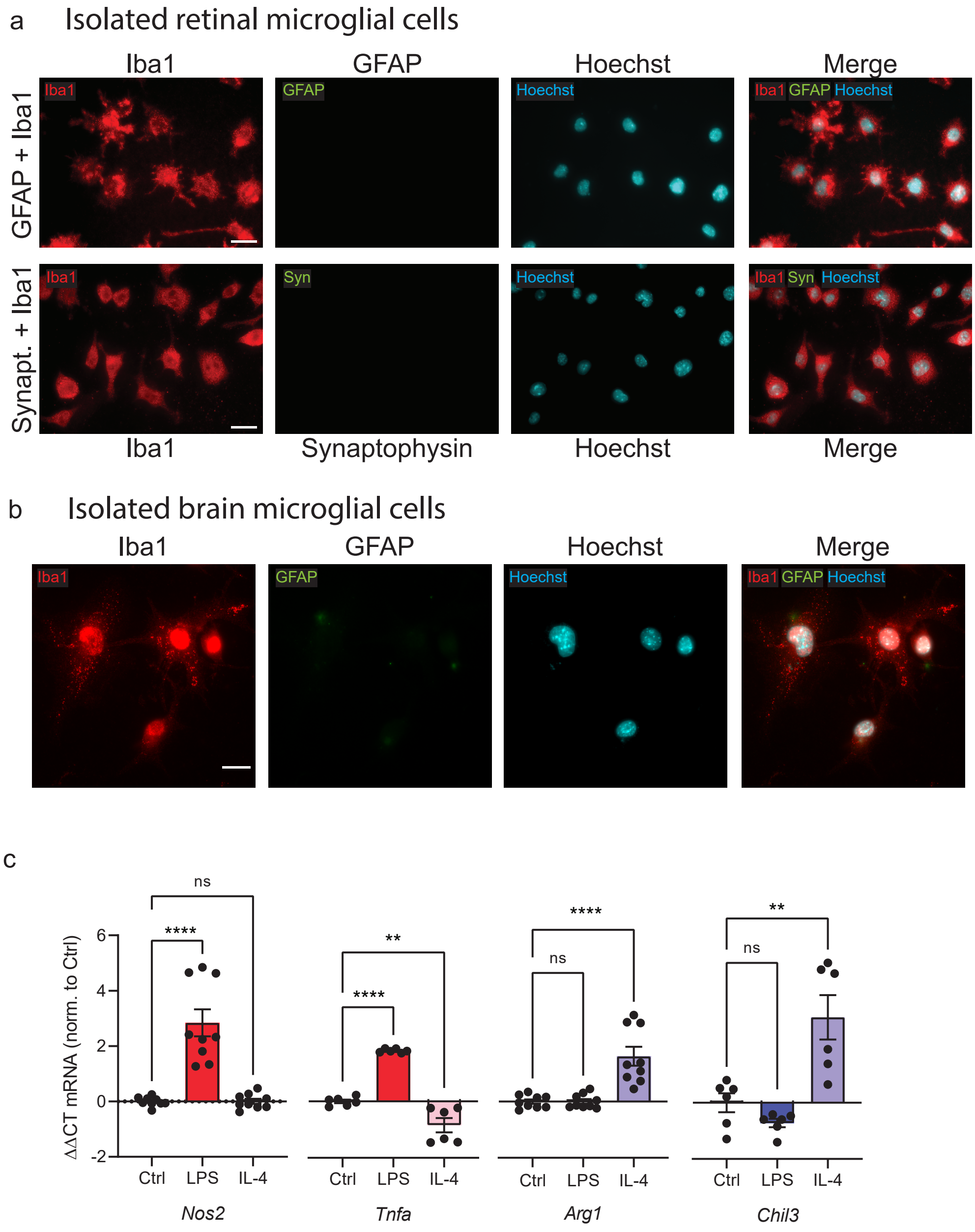

Supplementary Figure 5

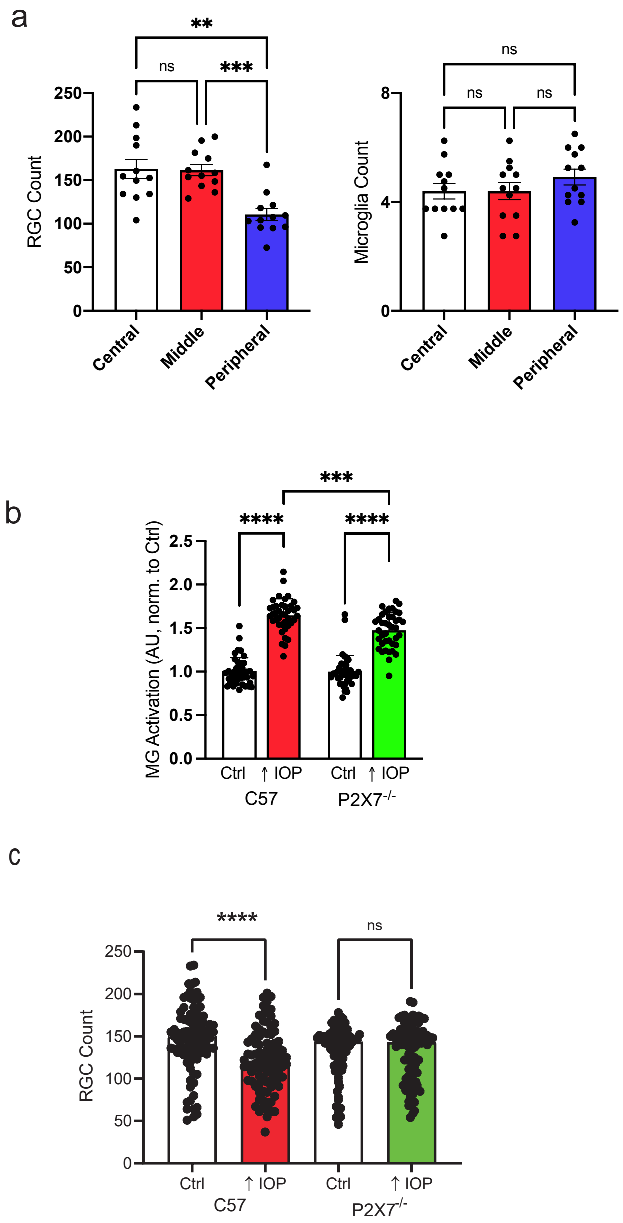

Supplement: Supplementary file 1 — Additional file 1 : Supplementary Figure S1. Details of effect of intravitreal injection of BzATP on Sholl and other image analysis. a Representations of i- Iba1 staining; ii- image tracing, iii - concentric rings placed every 5 μm for analysis of crossing and length; and iv - conversion to a binary image. Bar = 25 μm. b, c Tracings were analyzed for distance from soma at which peak number of intersections with concentric rings (b) and farthest intersection with concentric ring (c) occurred (paired Student’s t-test; n = 6 retinae of 3 mice). d Total summed branch length averaged per mouse was reduced by approximately 10% (paired Student’s t-test; n = 6 retinae of 3 mice; Normalized to mean saline injected = 1.0000, mean 250 μM BzATP = 0.8914). e Sholl analysis performed with individual cells (2-way Repeated Measures ANOVA with Sidak’s Multiple Comparison’s Test; n = 41 Saline, 46 BzATP) cells from 6 retinae of 3 mice; significance represents Multiple Comparison’s, data measured every 5 μm). f, g Absolute (f) and normalized (g, to avg saline) of Sholl data from individual images confirms reduction in branch length of microglia exposed to BzATP (unpaired Student’s t-test; n = 41 Saline, 46 BzATP cells from 6 retinae of 3 mice; Normalized mean saline = 0.9941, mean BzATP = 0.8930). h Quantification of Iba1 intensity in 5 μm ring around soma of individual cells from 6 retinae from 3 mice (unpaired Student’s t-test; n = 60 saline, 55 BzATP cells from 6 retinae of 3 mice). i. Schematic diagram indicating approximate placement of images analyzed for microglia and RGCs. *p < 0.05, **p < 0.01, ****p < 0.0001. Figure S2. Validation of observer scoring method of morphological microglial activation. a Representative images derived from Iba1-immunostained retinal whole mounts indicating a score of 1, 2, or 3. Bar = 50 μm. b A significant correlation between observer scoring and microglial Iba1-soma intensity was found. Observer scoring was normalized to the mean of 3 saline inje [file 12974_2021_2251_MOESM1_ESM.pdf]
