# Supplementary material for: Rapid morphologic changes to microglial cells and upregulation of mixed microglial activation state markers induced by P2X7 receptor stimulation and increased intraocular pressure
Source: J Neuroinflammation. 2021 Sep 20;18:217. doi: 10.1186/s12974-021-02251-7 (PMC8454080; doi:10.1186/s12974-021-02251-7)
Supplement: Supplementary file 2 — Additional file 2. : Supplemental Methods [file 12974_2021_2251_MOESM2_ESM.pdf]

Supplementary Methods

Intravitreal injections: Intravitreal injections were performed as previously described [1]. Briefly, after mice were anesthetized with 1.5% isofluorane, 1.5 µl Sterile Balanced Saline solution or 250 µM Benzoylbenzoyl-ATP (BzATP; Sigma Aldrich) was injected into the superior nasal region of the vitreous cavity approximately 0.5 mm from the limbus with a micropipette attached to a microsyringe (Drummond Scientific Co.). Animals were excluded if the lens was damaged. Differences in gene expression between saline-injected and naïve retinae from litter-control mates were not significant.

Immunocytochemistry: Isolated retinal microglial cells were mounted on 12mm glass coverslips coated with PLL and collagen as described in main text. Cells were fixed in 4% paraformaldehyde for 10 min at 37°C, washed in PBS with 1% Tween 20 (Bio-Rad), permeabilized with 0.1% Triton-X 100 for 15 min (Sigma-Aldrich) then blocked with 20% Superblock (Thermo Fisher) plus 10% goat or donkey serum. Primary and secondary antibodies used are listed in Table 1. After incubation in Hoechst (Cell Signaling, 1 µg/ml) for 10 min, coverslips were washed and mounted using SlowFade Gold (Thermo Fisher). Imaging was performed using a Nikon Eclipse microscope (Nikon) with NIS Elements Imaging software (Nikon v. 4.60). ImageJ was used in parallel processing to modify intensity, and merge pseudocolored images. Retinal cryosections were blocked with 1% Triton X-100, 0.5% Bovine Serum Albumin (BSA, Sigma-Aldrich), 0.9% Sodium Chloride (Thermo Fisher), and 5% donkey serum (DKS; Jackson ImmunoResearch) in 1% Phosphate-Buffered Saline (PBS-T-BSA), quenched using 0.3%, H_2O_2, then incubated with primary antibody overnight at 4°C, rinsed, then blocked in PBS-T-BSA. Secondary antibody was incubated for 2 hrs followed by application of 4′,6-diamidino-2-phenylindole (DAPI; 1:2000). Sections were

mounted using Fluoromount-G (Southern Biotech). Images were acquired from retinal cryosections, using a Nikon Eclipse microscope (Nikon, USA) and NIS Elements Imaging software (Nikon v. 4.60).

Image analysis for Sholl: Z-stacks were acquired from retinal whole mounts with a TCS SP8 confocal microscope (Leica). Iba1+ cells of the RGC and IPL layers were counted using FIJI [2] and randomized (www.randomizer.org). Fluorescence intensity was determined in a 5 µM radius of the nucleus center. Sholl analysis/summed branch length was performed outside of this radius using the FIJI Simple Neurite Tracer (SNT) plugin [3].

Time-lapse of morphological alterations: Retinal mouse microglia were plated and incubated in Mg^{2+}-free isotonic solution with 10 µM A839977 (Tocris) or DMSO as solvent control. Phase contrast images were taken every 12s or 15s using a Keyence BZ-X700 Series All-in-One Fluorescence Microscope (Keyence Corporation). BzATP (Sigma-Aldrich) was added in the presence of A839977 or vehicle. Representative video was derived using "Focus tracking" function, where time-lapse video is comprised of best-focused images derived from a panel of 7 images spaced 0.7 µm apart at each time point. FIJI [2] was used to modify intensity, with parallel processing for all time-lapsed sequences.

Observer quantification of morphological alterations: Retinal whole mount images were acquired from the central, middle, and peripheral regions of the retina as defined above using a Nikon Eclipse microscope (Nikon), with two images taken for each region in the superior, nasal, inferior, and temporal quadrants (Fig Si). De-identified images were scored individually based upon morphology. Smaller cell body size and elongated, thin processes received a score of 1, larger

body size and short, thick processes received a score of 3 (Fig. S2a). Observer scores of BzATP-exposed retinae correlated to measures Iba1-intensity of the soma region (Fig. S2b), and there was close agreement among observers, with the standard deviation of the scores between observers being less than 15% of the mean across all regions, validating the approach.

Transient elevation of IOP: Mice were deeply anesthetized with 1.5% isoflurane after receiving 2 mg/kg meloxicam. Proparacaine (0.5%) and tropicamide (0.5-1%) were administered and one eye was cannulated with a 30- gauge needle attached to polyethylene tubing (PE 50; Becton Dickinson) inserted into the anterior chamber, connected to a 20 ml syringe filled with sterile PBS. IOP was increased to 57.0 ± 0.4 mm Hg by elevating the reservoir to the appropriate height; blood flow through the retina was maintained throughout to avoid acute ischemia, although some reduction in blood flow was likely. After 4 hrs, IOP was returned to baseline, the needle removed, and 0.5% gentimycin was applied to the cornea. The contralateral eye without cannulation served as a normotensive control. Retinal tissues were isolated 22-24 hrs after elevation of IOP.

Sustained elevation of IOP: Sustained elevation of IOP was induced using the microbead injection method [4]. Mice were anesthetized in 2.5% isoflurane, and 2 μL of magnetic microbeads (COMPEL COOH-Modified 8-μm diameter; Bangs Laboratories) were injected into the anterior chamber of the eye using a glass-pulled micropipette connected to a manual microsyringe pump (World Precision Instruments) as described [5]. A neodymium magnet was used to draw the magnetic beads into the iridocorneal angle, blocking aqueous humor outflow through the trabecular meshwork and elevating IOP [4]. Both eyes were injected with beads to eliminate the

confounding factor of contralateral eye effects on glial activation [6]. Separate mice injected with saline served as controls. Minimum damage to ocular structures was observed. Ten IOP measurements per eye using a TonoLab tonometer (Colonial Medical Supply) were averaged; a baseline measurement was taken before bead injection, followed by weekly measurements after bead injection. The IOP integral (mm Hg-days exposure over baseline) was calculated to quantify cumulative IOP elevation [5]. Fixed retinae were cryoprotected in 30% sucrose (Sigma Aldrich) and 0.02% sodium azide (Sigma Aldrich) in 0.1M PBS and embedded in optimal cutting temperature medium. Sagittal sections at 10 to 15 µm using a Leica cryostat were obtained. Six to ten representative slides (three-four sections/slide) were imaged using a Nikon Eclipse microscope (Nikon). Mean intensity of Iba1 in microglia soma was analyzed using four sections per slide, and ten slides per retina using FIJI [2]. Data was derived from the central regions of the retina, consistent with above methods of intensity measurements.

References

1. Hu H, Lu W, Zhang M, Zhang X, Argall AJ, Patel S, et al. Stimulation of the P2X7 receptor kills rat retinal ganglion cells in vivo. Exp Eye Res. 2010;91(3):425-32 PMCID 2941978.
2. Schindelin J, Arganda-Carreras I, Frise E, Kaynig V, Longair M, Pietzsch T, et al. Fiji: an open-source platform for biological-image analysis. Nat Methods. 2012;9(7):676-82.
3. Longair MH, Baker DA, Armstrong JD. Simple Neurite Tracer: open source software for reconstruction, visualization and analysis of neuronal processes. Bioinformatics. 2011;27(17):2453-4.
4. Samsel PA, Kisiswa L, Erichsen JT, Cross SD, Morgan JE. A novel method for the induction of experimental glaucoma using magnetic microspheres. Invest Ophthalmol Vis Sci. 2011;52(3):1671-5.
5. Jassim AH, Inman DM. Evidence of Hypoxic Glial Cells in a Model of Ocular Hypertension. Invest Ophthalmol Vis Sci. 2019;60(1):1-15.
6. Rojas B GB, Ramírez AI, Salazar JJ, de Hoz R, Valiente-Soriano FJ, Avilés-Trigueros M, Villegas-Perez MP, Vidal-Sanz M, Triviño A, Ramírez JM. Microglia in mouse retina contralateral to experimental glaucoma exhibit multiple signs of activation in all retinal layers. J Neuroinflammation. 2014;11(133).
